# Developing and Evaluating a Data-Driven and Systems Approach to Health Promotion Among Vocational Students: Protocol for the Data Health Study

**DOI:** 10.2196/52571

**Published:** 2024-02-06

**Authors:** Clara Heinze, Rikke Dalgaard Hartmeyer, Anne Sidenius, Lene Winther Ringgaard, Anne-Louise Bjerregaard, Rikke Fredenslund Krølner, Steven Allender, Adrian Bauman, Charlotte Demant Klinker

**Affiliations:** 1 Department of Prevention, Health Promotion and Community Care Copenhagen University Hospital - Steno Diabetes Center Copenhagen Herlev Denmark; 2 National Institute of Public Health University of Southern Denmark Copenhagen Denmark; 3 Steno Diabetes Center Zealand Holbaek Denmark; 4 Institute for Health Transformation Deakin University Melbourne Australia; 5 School of Public Health Sydney University Sydney Australia

**Keywords:** health promotion, health behavior, well-being, organizational readiness, cocreation, causal loop diagram, systems thinking, systems-based evaluation, vocational schools, youth

## Abstract

**Background:**

Vocational school students exhibit significant risk behaviors in terms of poor diet, frequent use of nicotine products, inadequate fruit and vegetable intake, low levels of physical activity, and poor mental health. This makes vocational students vulnerable to the development of noncommunicable diseases. Therefore, effective health promotion programs targeting vocational students are required.

**Objective:**

The Danish study “Data-driven and Systems Approach to Health Promotion Among Vocational Students” (Data Health) aims to develop, implement, and evaluate a systems approach to support vocational schools, municipalities, and local communities in implementing locally relevant health promotion actions among and for vocational students. This paper describes the Data Health program and how implementation and preliminary effectiveness will be evaluated.

**Methods:**

The Data Health program offers an iterative 5-step process to develop changes in the systems that shape health behavior and well-being among vocational students. The program will be implemented and evaluated in 8 Danish vocational schools in 4 municipalities. The implementation of the process and actions will be explored using a systems-based evaluation design that assesses contextual differences and the mechanisms through which the program leads to changes in the systems. Preliminary effectiveness at the individual level (students’ self-reported health behavior and well-being) and organizational level (school organizational readiness reported by school staff) will be assessed using a quasi-experimental design, and cross-sectional data will be collected at all 8 schools simultaneously 4 times during the 2-year study period.

**Results:**

This study was launched in 2021, and data collection is expected to be completed in June 2024. The first results are expected to be submitted for publication in January 2024.

**Conclusions:**

We expect that the Data Health study will make significant contributions to complex intervention research by contributing to the paucity of research studies that have used systems approaches in school settings. The study will also provide evidence of successful elements for systems change and effectiveness to determine whether a national scale-up can be recommended.

**Trial Registration:**

ClinicalTrials.gov NCT05308459; https://clinicaltrials.gov/study/NCT05308459

**International Registered Report Identifier (IRRID):**

DERR1-10.2196/52571

## Introduction

### Social Inequalities in Health

Globally, 71% of all deaths are due to noncommunicable diseases (NCDs), and more comprehensive preventive strategies are needed to reduce NCD mortality worldwide [1]. Social gradients exist in NCD risks (tobacco use, physical inactivity, unhealthy diet, and harmful use of alcohol) and NCD-related premature deaths. Populations with higher educational attainment have better health than those in lower socioeconomic groups [2]. Most students attending vocational schools come from low-income families [3,4], and an ordinary vocational degree is defined as “shortened school education.” This makes vocational students vulnerable for NCDs, and already as students, they report a high prevalence of tobacco smoking, inadequate fruit and vegetable consumption, physical inactivity, and obesity [5]. For example, in Denmark, 39% of vocational students are overweight (BMI >25 kg/m^2^) compared with 14% of high school students, and this prevalence among vocational students is increasing (2014: 30%; 2019: 39%) [6,7]. Despite this, students in vocational education are an often overlooked but important group to target for health promotion to reduce health inequalities [5].

### The Complexity of Health Behavior

The application of health promotion can strengthen the capability of individuals to take action and build the capacity of groups, communities, or organizations to act collectively to exert control over the determinants of health [8]. The determinants of health are complex and influenced by underlying dynamic factors at multiple levels (ie, individual, intrapersonal, organizational, environmental, neighborhood, and structural) [9,10]. Evidence suggests that chancing organizational structures and processes and strengthening relationships between organizations are more likely to maintain healthy behavior among individuals than programs that target individual behavior change only [11]. In addition, multicomponent approaches (a combination of structures, processes, and individual behavior changes) for health promotion are more effective than single-component programs [12,13].

A challenge for health promotion is the “voltage drop” that has been identified when effective programs are scaled up across contexts (eg, schools of different sizes), as different contexts lead to different outcomes [14,15]. Vocational students’ health behaviors and well-being vary across educational tracks (technical, business, agriculture and food services, social and health services) and regions [7], requiring the use of local data to identify intervention opportunities and priorities. Data-driven programs that use relevant local data to strengthen intervention design are emerging in health promotion research. These programs explicitly consider local conditions as a means of increasing the strength and relevance of the interventions implemented [16,17].

### Approaches to Health Promotion in Schools

Schools are important settings that influence health behaviors [18]. In the Danish vocational school reform in 2015, vocational schools were mandated to engage in health promotion to increase students’ healthy behavior and well-being and reduce dropout [19]. There is little evidence on how to support vocational schools to implement effective and sustainable health promotion. Vocational schools have struggled to implement and integrate health promotion into their core business [20], so more knowledge is needed about what approaches are feasible, effective, and sustainable in this setting. In Denmark, the smallest administrative unit, the municipalities, are responsible for health promotion among all citizens within their jurisdiction [21]. This places the 92 Danish municipalities as central to vocational school health promotion, but many schools and their students often cross municipal boundaries, making the responsibility for vocational school health promotion unclear and therefore less of a priority in many Danish municipalities [21]. However, previous research shows that municipalities are keen to work with health promotion at vocational schools but often lack a framework for these activities [22].

The World Health Organization’s Health Promoting Schools (HPS) principles [23] state that efforts to improve health should go beyond individual behavior change to consider local contexts, organizational and policy changes (eg, from educating individuals to choosing alternative foods to changing the foods available), or a combination of these [14]. In addition, the HPS principles argue that involving the surrounding community and key community leaders will create a more sustainable environment that can support the maintenance of healthy behavior changes among students [24]. Systems thinking has emerged as a method for understanding and changing the drivers of complex health behavior challenges [10,25]. A systems perspective considers a broad approach that engages different groups of participants to contribute to the design, implementation, and evaluation of actions that disrupt current systems and normalize change [10,26]. Identifying and activating modifiable parts of systems (ie, leverage points) is essential to change structures, goals, and beliefs in the systems and thus achieve sustainable health behavior change [27]. Engaging key stakeholders in coordinated efforts [28] has been conducted in recent attempts to apply systems approaches within health promotion (eg, obesity [17,29-31], reducing dietary inequalities [32] and physical inactivity [33], and tobacco control [34]). In combination, HPS approaches and systems thinking provide a set of methods and perspectives that could be effective and sustainable for school health promotion [35].

### Objectives

The Data-Driven and Systems Approach to Health Promotion Among Vocational Students (Data Health) study aims to develop, implement, and evaluate a systems approach informed by local data to support vocational schools, municipalities, and local communities in implementing locally relevant health promotion actions among and for vocational students. This protocol describes the Data Health program and how its implementation and preliminary effectiveness will be evaluated at 8 vocational schools across 4 municipalities in Denmark to determine if national scale-up can be recommended.

## Methods

### The Vocational School Setting

Vocational education in Denmark prepares students for specific occupations and is divided into four main educational tracks: (1) technical (eg, electrician), (2) business (eg, office assistant), (3) agriculture and food service (eg, farmer or chef), and (4) social and health service (eg, health care assistant). Vocational education alternates between school-based training and workplace-based training, with approximately one-third of the time spent in school [36]. Some schools are multisited and have student populations greater than 6000, whereas other schools are located at a single address with a student population of no more than 100. Almost two-thirds of students are men (64.7%), but the gender distribution varied according to educational track; for example, the proportion of men is 89.3% in technical education but only 12.3% in social and health service education [37]. The average age of students is 24 years; some students enroll directly from primary school (aged 15-17 years), whereas others begin later in adult life (39.8% is aged >25 years) [37].

### Recruitment

We will use a purposive sampling strategy. A total of 8 vocational schools will be recruited based on the region (Capital Region of Denmark and Region Zealand) and educational track (technical, business, agriculture and food service, and social and health service) to include one vocational school from each educational track in both regions. Simultaneously, the municipalities where the vocational schools are geographically located will be recruited. The enrolled schools and municipalities will be responsible for recruiting community actors with support from the research team, if needed. The role and responsibilities of the actors in the community is described in steps 3 and 4 of the program. In this case, “community actors” refers to individuals, organizations, or companies, both local and national, who can provide resources and social support for the implementation of actions for change.

### Content of the Data Health Program

#### Development

**Figure 1:**
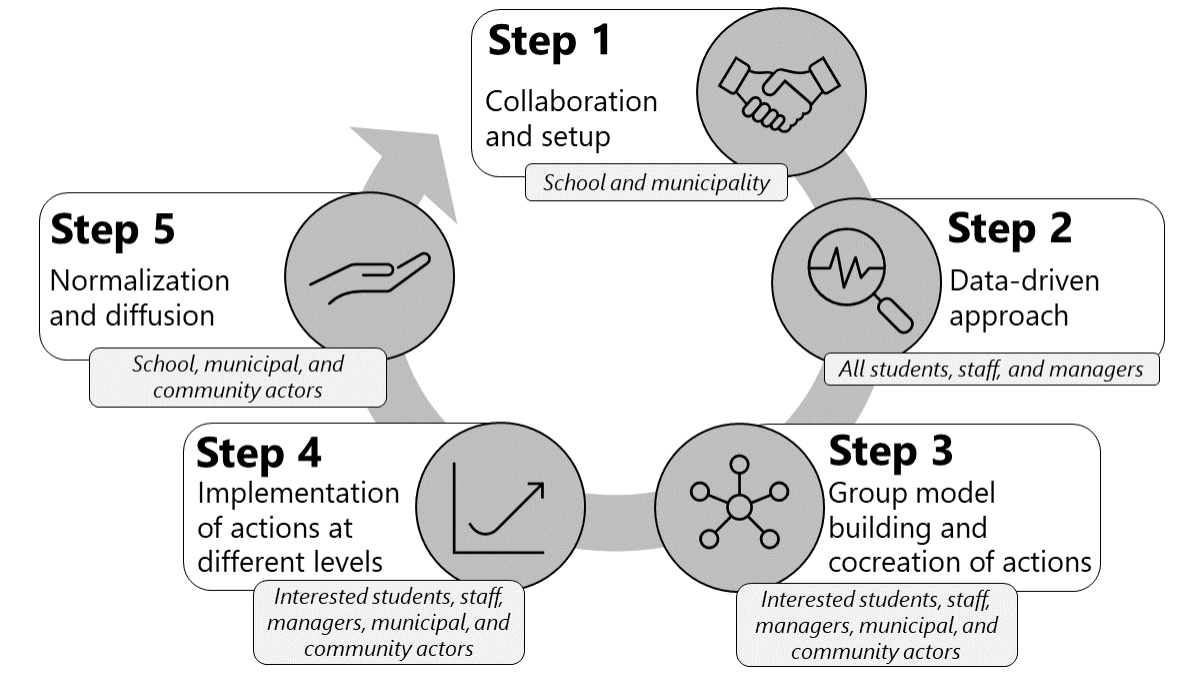
The 5 steps of the Data Health program.

The Data Health program offers an iterative 5-step process (Figure 1) for developing change in the systems to shape vocational students’ health behavior and well-being, combining best practices in health promotion research, including methods from systems science [10,38], data-driven approaches [16,39], and the World Health Organization’s HPS principles [23]. The program was developed by a health promotion research group piloted and formatively evaluated in 1 vocational school and 1 municipality from June 2021 to June 2022. The formative evaluation included observations and interviews and surveys of key participants from both school and municipality. Weekly meetings were held between the evaluator (AS) and the research team to discuss the experiences, barriers, and enablers of implementation. During the formative evaluation, suggestions for program changes were made by exploring the municipality and vocational school environmental contexts and how the program would fit or function within these settings. The research team adjusted and incorporated changes to the program steps throughout the pilot test.

The research team will be responsible for the implementation of steps 1 to 3 of the program but will concurrently build the capacity, motivation, and commitment of the school and municipal program coordinators to be responsible for the implementation of actions and to promote the normalization of the program (steps 4-5).

#### Step 1: Collaboration and Setup

The Data Health program will begin with the establishment of a formal collaboration agreement between management representatives from the vocational school, the municipality, and the research team. Both the school and the municipality will appoint 1 or 2 staff members as school and municipal program coordinators, respectively. One month of salary will be provided to each school program coordinator (€4000; a currency exchange rate of €1=US $1.09 is applicable) to support program implementation. The roles and responsibilities of the different partners will be clarified and negotiated. Once the collaboration and commitment at the management level has been secured, information about the program will be disseminated by the school management to all staff and students. Dissemination activities include information by email and a kick-off meeting for staff, whereas dissemination for students includes customized posters and flyers distributed in common areas of the school. On the basis of the learnings from the pilot study, we have enhanced the dissemination strategy for the program to boost program visibility at the schools and comprehension among both staff and students.

#### Step 2: Data-Driven Approach

Local survey data on students’ health behaviors and well-being will be collected and analyzed by the research team (see the description of individual outcomes in research question [RQ] 5). To stimulate motivation and interest, the data will be returned to the school and municipality within few weeks in the form of a local health profile. The local health profile covers modifiable health risk factors among vocational students, for example, nicotine use, unhealthy eating habits, physical inactivity, and poor mental health [7]. A simplified version of the health profile will be presented by the research team and the municipal program coordinator at separate meetings for school managers and staff and for students to share and increase their knowledge about health promotion, stimulate discussions and reflections about the results, and promote engagement and motivation for change. On the basis of the data presentations and discussions, the staff and students will be asked to complete a short questionnaire to select the health issue they are most motivated to address. During the pilot, we explored how best to select the specific health issue to be addressed in the remaining steps of the program and who should make this decision. The conclusion was that it is a school management decision, but schools are strongly encouraged to include the perspectives of staff, students, the municipality, and data from the local health profile in the process. To increase the prospect of becoming an iterative model, the local health survey has been designed to match an existing municipal-based health profile system [40] that the school-municipal collaboration can sign up after the research team has withdrawn the program.

#### Step 3: Group Model Building and Cocreation of Actions

Group Model Building (GMB) is a participatory method from systems science to facilitate a shared understanding of the structures and relationships that shape the system through the creation of causal loop diagrams (CLDs) [38,41]. A CLD helps to build a shared understanding among participants of the cause and effect relationships within a given system and to identify and agree on relevant and important areas for change [41]. In previous community research, this approach has been shown to improve the understanding of the problem, develop consensus on actions for change among a diverse group of participants, and increase participants’ social network [41,42].

GMB is a central part of the Data Health program, as we aim to involve and engage a wide range of participants in developing a CLD and identifying locally adapted actions for change. In this program, the GMB process consists of 3 sessions (GMB1-3), as proposed and applied by an Australian research group at the Institute for Health Transformation, Deakin University [43]. The Data Health research team was trained in the GMB process by this group and then adapted the process and methods to a vocational school setting and vocational students, primarily based on the approaches and results of the pilot test.

In GMB1-2, participants will cocreate a CLD [44] to gain a shared understanding of the perceived causes and drivers of the health issue selected in step 2, for example, poor mental health or physical inactivity. Participants will also identify existing initiatives or programs in the school or community that could have an impact on perceived causes and drivers. GMB1-2 will involve 5 to 10 students, 5 to 10 school staff members, the school and municipal program coordinators, and school management. The school program coordinator will recruit a diverse group of motivated students and staff based on gender, age, and education. The Systems Thinking in Community Knowledge Exchange computer software [45] will be used during this process. At the end of GMB2, participants will identify several community actors who are considered relevant or necessary for the identification and implementation of actions across the school, community, or municipality. These community actors, representing different organizations, sectors and areas of expertise, will then be invited to GMB3 via phone calls. Together with the participants from GMB1-2 and other interested students and staff members, this wider group of participants will identify leverage points in the CLD and prioritize actions to change the system. Within systems thinking, actions can range from minor actions to major actions [27,41]. Minor actions often aim to solve a single issue and can often be implemented quickly with low resource costs (eg, single events), whereas major actions can aim to change paradigms in the way individuals or organizations think and behave and are often more difficult and costly to implement (eg, changes in an organization’s goals and beliefs) [27]. The principle in systems thinking is that minor actions can stimulate major actions [27,41].

Participants will identify existing actions or codevelop new actions to change specific elements of the system or the entire system. Actions for further implementation will be selected based on feasibility and expected impact. As a result of GMB3, “action groups” of community actors, staff, and students will be formed, based on motivation, to plan and implement one or more specific actions.

#### Step 4: Implementation of Actions at Different Levels

In the months following GMB3, the action groups will plan and implement actions for changing the system, assisted by the school and municipal program coordinators. Examples of actions at different levels can be found in the description of program evaluation (implementation of actions [RQ2]). To support the coordinators and action groups, a guide to the development and implementation process has been adapted from previous work [38]. In addition, the Data Health study has reserved funds for each school (€1350) to support the implementation of actions or involvement of community actors to run implementation.

#### Step 5: Normalization and Diffusion

The school and municipal program coordinators will be responsible for the continuation of the program, and the resources available for this will depend on the priorities of these organizations. The research team will organize meetings between relevant community actors and managers from the school and municipality to encourage the initiated collaborations to remain formalized beyond the program. At these meetings, we will develop a strategy for the continuation of the actions already initiated and a plan for new data collection and development of new local actions. Most municipalities in Denmark have a questionnaire tool [40] for data collection to support the municipalities’ work with health behavior and well-being among children and youth. The municipalities will be encouraged to use the tool for future data collection at the program school, as well as at other vocational schools within the municipality.

To increase and maintain momentum for health promotion practices among vocational students, all school and municipal program coordinators and relevant community actors will connect across study sites every 4 months in a community of practice (CoP), initiated by the research team. The CoP is a forum for sharing experiences and learnings on effective strategies for health promotion and collaboration across study sites but will eventually be open to other schools and municipalities interested in implementing the Data Health program.

If all 5 steps are completed as intended, we expect various elements of the Data Health program to be sustained, including the actions implemented, the collaborations initiated, and the organizational practices and motivation to repeat the monitoring to evaluate changes and develop new actions.

### Program Evaluation

#### Research Questions

In total, 5 RQs will comprise the evaluation of the Data Health program:

- RQ1 (process evaluation—steps 2 and 3 of the program): To what extent is the data-driven approach and the GMB process implemented as intended, and what seem to be the most important mechanisms of change and contextual factors?
- RQ2 (implementation of actions—step 4 of the program): What characterizes the planned and initiated actions and what are the unintended consequences related to implementation? Who is involved in the planning and implementation?
- RQ3 (program normalization—step 1 and 5 of the program): What are the opportunities, barriers, and needs for the collaboratives and program to be sustained and normalized?
- RQ4 (organizational outcomes): Does the program stimulate organizational changes in schools to work in a more health-promoting direction?
- RQ5 (individual outcomes): Does the program contribute to improvement in health behaviors and well-being among vocational students?

#### Study Design

To evaluate the implementation of the program, related actions, and systems impact (RQ1-3), we will use a systems-based evaluation design that seeks to understand how the program and the systems adapt to each other [10,46]. Evaluation using a systems perspective needs to adapt as the program unfolds by examining emergent outcomes that result from the interactions of participants [10,46], and it involves examining relationships, interactions, and patterns rather than individual outcomes and static “snapshots” [46,47]. The systems-based evaluation design aims to gain an in-depth understanding of the system as a whole without a “control system.” However, it is not possible to include every part of a dynamic system; therefore, it is necessary to define systems boundaries to determine what is considered relevant in terms of what and where to evaluate [46,48]. The starting point for defining the boundaries is the identified primary health issue that each of the 8 program schools has chosen to target. All organizations and interventions relevant to the targeted problem will be considered as part of the system. In terms of system factors, we will focus on modifiable social and physical environmental factors rather than psychological or genetic factors.

**Figure 2:**
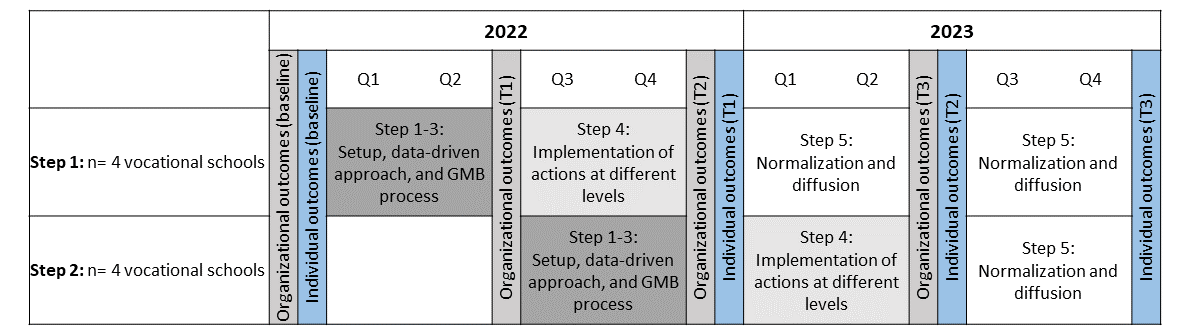
Study design and timeline of the data collection for the effectiveness evaluation. GMB: Group Model Building.

We will examine preliminary effectiveness (see descriptions of organizational outcomes [RQ4] and individual outcomes [RQ5]) at the organizational level (ie, school organizational readiness) and at the individual level (ie, students’ health behavior and well-being) using a quasi-experimental design with a nonrandomized clustered stepped-wedge strategy. The 8 program schools will be enrolled in 2 steps, matched by educational track (one school from each main track) and geographic location (2 schools from 2 Danish regions). The matched clusters will be assigned to early start (January 2022) or late start (6 months later). This design allows for a sequential roll-out of the program and allows us to control for differences between study sites (vertical control) and secular trends (horizontal control) during the study period [49]. Organizational- and individual-level data will be collected simultaneously at all 8 schools 4 times during the 2-year study period at baseline and at 3 follow-up points (T1, T2, and T3; Figure 2). As systems change takes time to diffuse into individual behavior changes [31], the research team will in due course seek opportunities and funding for longer-term data collection (ie, a 5- and 10-year follow-up).

#### Program Theory

**Figure 3:**
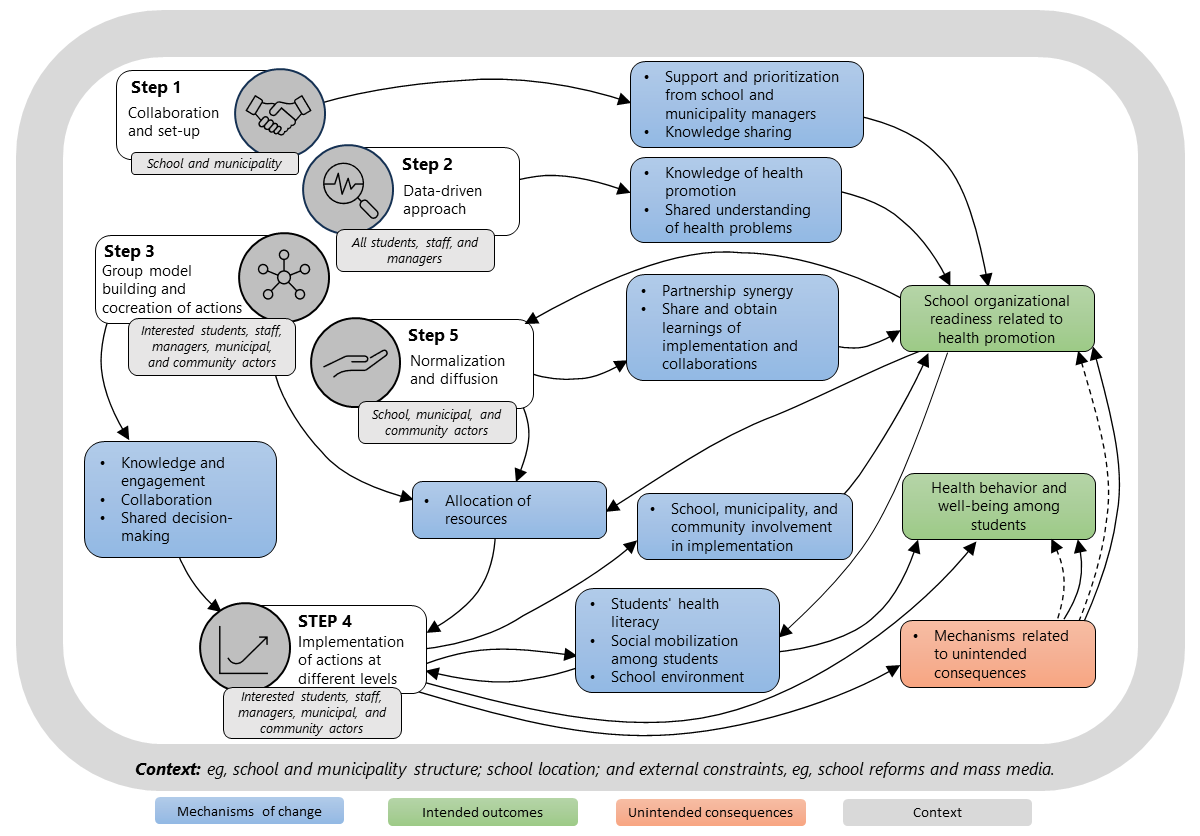
The Data Health system map.

A program theory explains how a program is expected to work and under what conditions [10]. The Data Health program theory (Multimedia Appendix 1) is captured in a series of “if-then” statements and is based on key findings from the pilot test, published literature reporting trial results or theoretical abstractions [8,16,27,50-53], and the research group’s experiences and previous research in the field [20,54-57]. Mechanisms of change are the hypothesized causal links between the program components and identified outcomes, triggered within the contexts in which the program is implemented [10]. In total, we hypothesize 6 mechanisms of change as plausible causal links between the data-driven approach and the GMB process and outcomes; these are indicated by mechanisms connected to steps 2 and 3 in Figure 3. To understand the interactions among context, program components, mechanisms, and outcomes; the research group visualized the initial program theory as a systems map, illustrated in Figure 3. The map summarizes our proposed model of how program components are expected to lead to systems change and outcomes. The connecting arrows show how changes in one part of the system are expected to generate changes in other parts of the system. The Data Health program theory and system map will continually be refined and revised as part of the evaluation process.

#### Process Evaluation (RQ1)

In the process evaluation [58], we will examine whether the data-driven approach and the GMB process (steps 2 and 3 of the program) are being implemented as intended, the associated contextual factors and the mechanisms of change.

Both quantitative and qualitative methods will be used to collect data for the process evaluation (Table 1). Following the Medical Research Council guidelines [58], 5 process evaluation components will be assessed: recruitment, reach, fidelity, dose delivered, and dose received. In addition, we will examine whether the 6 hypothesized mechanisms of change are activated and the extent to which these mechanisms are modified through their interaction with contextual factors. However, we will remain open to other emerging mechanisms.

**Table 1:** Items, methods, instruments, and frameworks used in evaluation of implementation (research questions 1-3).

Component and items of interest			Methods and data	Applied evaluation instruments and frameworks
**Process evaluation—steps 2 and 3 of the program**				
	**Context information**			
		Examples: School type School size School location School and municipal structure	Semistructured interviews with the principal school managers and the municipal program coordinators (after GMB^a^3) Facts obtained through school and municipality websites	Medical Research Council guidance [58]
	**Implementation**			
		What is delivered: Recruitment Reach Fidelity Dose Adaptations	Registration of participants during meetings and GMB sessions Exit surveys to all participants (after data presentation meetings, GMB2 and GMB3), semistructured interviews with the school program coordinators (after GMB2) and the principal managers (after GMB3)	Medical Research Council guidance [58] COMPACT Stakeholder-driven Community Diffusion Survey [59]
	**Mechanisms of change**			
		Knowledge of health promotion Shared understanding of health issues Motivation and engagement Collaboration Shared decision-making Resource allocation Other emerging mechanisms	Exit surveys to all participants (after data presentation meetings, GMB2 and GMB3), semistructured interviews with the school program coordinators (after GMB2) and the principal managers (after GMB3)	Medical Research Council guidance [58] COMPACT Stakeholder-driven community diffusion survey [59]
**Implementation of actions—step 4 of the program**				
	**Actions**			
		Number of actions initiated Primary variable of influence Action level (beliefs, goals structures, events)	Semistructured interviews with the school and municipal program coordinators quarterly	The Systems Thinking in Community Knowledge Exchange computer software [45] Action Scale Model [27]
	**Involvement**			
		Number of involved participants Level of involvement in preparation, execution, and implementation of the developed actions	Semistructured interviews with the school and municipal program coordinators quarterly	The involvement matrix [53]
	**Unintended consequences**			
		Rippling effects and potential positive and negative unintended consequences of the actions implemented	The community of practice network will be involved in a process (meetings or workshops) of identifying the rippling effects	Ripple effect mapping [60]
**Program normalization—steps 1 and 5 of the program**				
	**Maintenance of collaboratives**			
		Potentials, barriers and needs Partnership synergy	Notes from the meetings for program maintenance (6 months after GMB3) Semistructured interviews with the school manager (after GMB3) and the municipal program coordinator managers (6 months after GMB3)	Partnership synergy [51]
	**Program normalization**			
		Coherence Cognitive participation Collective action Reflexive monitoring	Semistructured interviews with the school manager (after GMB3) and the municipal program coordinator managers (6 months after GMB3)	Normalization process theory framework [61]

^a^GMB: Group Model Building.

Qualitative and quantitative data will be interpreted separately and combined. Quantitative data will be analyzed descriptively, whereas qualitative interviews will be recorded, transcribed, and thematically analyzed in NVivo (Lumivero) [60].

#### Implementation of Actions (RQ2)

Actions initiated by the school, municipal, or community actors and the consequences of these actions are a direct indicator of systems change. The number of actions initiated and the primary factors in the locally developed CLD that are influenced will be tracked and visually added to the CLD. The Action Scale Model [27] will be used to determine whether the actions address one of four hierarchical levels to change the functioning of the system in the anticipated direction: (1) beliefs (eg, the school creates a local action group as health promotion champions), (2) goals (eg, the school sets new goals for health promotion), (3) structures (eg, school and municipal staff receive training on the complexity of health), and (4) events (eg, a single sport event at schools) [27]. The hierarchical structure suggests that changing beliefs and goals will not only have a greater systemic impact but also be more costly and time consuming than implementing single events [27]. We will also examine which participants (ie, students, school staff and management, municipal staff, and community actors) are involved in the planning and implementation of the actions and how and to what extent they are involved. The involvement levels of the participant groups will be interpreted using the involvement matrix [53].

All actions and involvement will be tracked on a quarterly basis for up to 2 years after GMB3 through semistructured interviews with the school and municipal program coordinators and involved community actors to enable interpretation of the level of system change and engagement across participant groups over time (Table 1).

In addition, an adapted version of “ripple effect mapping” [62] will be used to understand the rippling-initiated actions, effects, and potential positive and negative unintended consequences of the actions implemented. The CoP will be involved in a process to explore and visualize unintended consequences.

#### Program Normalization (RQ3)

We will explore the opportunities, barriers, and what is needed to integrate the collaboratives and the program into normalized practice (steps 1 and 5 of the program).

The school-municipality collaboration and program normalization will be explored through interviews with school and municipal program coordinators (Table 1). Interview guides inspired by the Partnership Synergy framework [51] will be developed to explore the functioning of the collaborations and the potential for maintenance, and the normalization process theory [61] will be used as a framework for understanding program normalization. In addition, carefully written notes will be taken from the program maintenance meetings, which will be completed as part of step 5 of the program.

Interviews will be recorded and transcribed, and all data will be analyzed thematically in NVivo [60] to understand opportunities, barriers, and needs related to sustaining collaborations and normalizing the program. Finally, we will develop recommendations for the implementation of systems approaches for sustainable health promotion in vocational schools.

#### Organizational Outcomes (RQ4)

School organizational readiness to address health promotion is selected as a primary organizational outcome. We will apply the “organizational readiness framework” [50] to track change over time. Organizational readiness refers to the extent to which an organization is willing and able to implement change, and the elements in the framework have previously been shown to be related to implementation success [63]. School organizational readiness will be assessed by questionnaires administered to all school staff, supplemented by semistructured interviews with the principal managers at the schools (Table 2).

**Table 2:** Items, methods, instruments, and frameworks used in evaluation of effectiveness (research questions 4 and 5).

Component and items of interest				Methods and data		Applied evaluation instruments and frameworks
**System outcomes**						
	**Organizational readiness**					
		Baseline and change in the following: Organizational motivation General capacity Health promotion capacity	Questionnaire to staff members (4 times in total) Semistructured interviews with principal managers (after GMB^a^3)		Organizational readiness [50]	
**Individual outcomes**						
	**Health behavior**					
		Baseline and changes in the following: Mental health and well-being Physical activity Food intake Nicotine use Alcohol use	Questionnaire to all students (4 times in total)		WHO-5^b^ [64] and SGPALS^c^ [65] Validated measures from the Danish National Health Survey and the Health Behaviour in School-aged Children study [66]	
	**Health promotion outcomes**					
		Baseline and changes in the following: Health literacy Knowledge relevant to the problem of interest Self-empowerment Self-confidence Self-efficacy Behavioral intentions Motivation Social network and interaction Social connectedness Student autonomy	Questionnaire to all students (4 times in total)		HLQ^d^ [67]; HLSAC^e^ [68] validated measures from the Danish National Health Survey and the Health Behaviour in School-aged Children study [66]	

^a^GMB: Group Model Building.

^b^WHO-5: World Health Organization (Five) Well-Being Index.

^c^SGPALS: Saltin-Grimby Physical Activity Level Scale.

^d^HLQ: Health Literacy Questionnaire.

^e^HLSAC: Health Literacy for School-Aged Children.

The questionnaire instrument will adapt and refine existing and tested items [20,69] across 3 dimensions and 10 subdimensions: motivation (relative advantage, compatibility, complexity, and priority), general capacity (culture, climate, and staff capacity), and program-specific capacity (knowledge, skills, and abilities). We will use exploratory and confirmatory factor analyses to initially validate the instrument and assess its internal consistency and convergent validity. The questionnaire will be distributed electronically via email in SurveyXact [70] to all school staff (eg, teachers, counselors, and administrators), and repeated data will be collected 4 times during the study period (Figure 2). Changes in school organizational readiness will be assessed primarily using linear mixed modeling, adjusting for clustering, with school as a random effect and time as a fixed effect.

Interviews with school leaders will contribute to a deeper understanding of how the support and priorities of the school management can stimulate or hinder the school’s readiness and willingness to change. The interviews will be recorded, transcribed, and analyzed thematically according to the dimensions and subdimensions of organizational readiness.

To generate a comprehensive understanding of school organizational readiness, a convergent parallel mixed methods design will be used in the analyses [71]. Quantitative and qualitative data will be analyzed independently and then compared, related, and interpreted.

#### Individual Outcomes (RQ5)

We will explore the indications of effectiveness at the individual level through changes in students’ health behaviors and well-being. Items to assess health behavior will primarily consist of validated measures and items used in other national health profile studies in Denmark (Table 2). The research team will preidentify one primary and one secondary outcome indicator item for each targeted health area (ie, dietary behavior, physical activity, alcohol consumption, marijuana and drug use, and well-being). The primary indicator will be an item assessing students’ health behavior during school time, and the secondary indicator will be an item assessing health behavior during leisure time or overall.

Following Bauman and Nutbeam [8], we will assess both health behavior and health promotion outcomes to understand the complexity of health behavior. While health behavior outcomes are expressed in terms of changes in, for example, levels of physical activity or mental well-being, health promotion outcomes are personal, social, and environmental factors that are a means of improving people’s ability to change behavior [8]. Therefore, health promotion outcomes are considered intermediate to health behavior (see Table 2 for examples).

The questionnaire data will be collected 4 times (Figure 2). Regardless of the specific health issue each school chooses to address, all students will receive the same questionnaire to identify potential intended and unintended consequences on other health promotion outcomes or behaviors, as suggested in a complex systems sense [46,72]. Electronic questionnaires in SurveyXact [70] will be distributed during school hours by the research team, who will be present in the classrooms so that students can ask questions as they complete the questionnaire. The estimated student population of the 8 program schools is approximately 2000 and, based on previous experience with this procedure [54], we expect that 90% of the students present in class will complete the questionnaire at each of the 4 data collection points. Informed consent will be obtained from all students and can be withdrawn at any time.

Most analyses will be based on cross-sectional data and descriptive in nature, interpreting the mean differences between the intervention and control groups. However, the analyses will also include linear mixed models with time and selected covariates (eg, gender, age, ethnicity, and socioeconomic status) as fixed effects and school as a random effect. It is important to note that the total time of 2 years to assess individual outcomes may be too short to provide conclusive evidence at this level.

### Ethical Considerations

The study has been referred to the Capital Region of Denmark’s legal center for personal data handling (journal number: P-2021-327). The study will be conducted without approval from the Committees on Health Research Ethics for the Capital Region of Denmark (journal number: 22012766), as this is not required for social health science in Denmark [73]. Participation in research related to the study is voluntary and requires written informed consent from all participants. Consent may be withdrawn at any time. Questionnaire data, key files, audio files, and transcripts of interviews and observations will be stored in a secure folder on the corporate network in accordance with the requirements of the Capital Region of Denmark and European General Data Protection Regulation. Only the principal investigator and those with permission from the principal investigator will have access to data. None of the authors have any financial or competing interests.

## Results

This study was launched in 2021, and data collection is expected to be completed in June 2024. The first results will be submitted for publication in January 2024. The results of this research will be disseminated through national and international conferences, peer-reviewed journals, reports, and web-based sources. In addition, key findings will be disseminated through the CoP and other national practice network meetings with stakeholders and policy representatives.

## Discussion

### Overview

The Data Health study is the first to apply a systems approach to the implementation and evaluation of health promotion programs among vocational school students. The program comprises 5 steps aimed at establishing strong collaborations, building local capacity, identifying leverage points, and generating and implementing collective actions for systems change to improve health and well-being among vocational students. The program will be accompanied by a systems-based evaluation to support the sustainability of program implementation and to determine the scalability of the program. Guided by the Medical Research Council guidance, the evaluation will assess implementation outcomes, contextual differences, and the mechanisms through which the program leads to changes in systems, school organizational readiness, and health behavior and well-being.

### Strengths and Limitations

#### Program Implementation

The steps of the Data Health program are based on systems and health promotion theory, previous health promotion evidence, and formative evaluation at a pilot site. This study has the potential to improve the health behavior and well-being of vocational students and reduce social inequalities in health. The use of systems approaches in communities to address childhood obesity has shown promising results [31,74]. However, adaptation to disadvantaged educational settings may be challenging. One possible challenge is to engage students who are only in school for short periods between workplace-based training. As workplaces are likely to be located across municipalities, it may be difficult to engage students in the GMB process and implementation of actions during workplace training. If possible, we will try to involve the local workplaces in creating collective actions in GMB3 to involve the students, even if they are not physically at the school.

In most health promotion programs, resources often run out before the desired changes in individuals or systems are normalized [75]. The sustainability of actions for system change has been demonstrated in communities, with actions continuing to be implemented years after the reduction in research support [76]. On the basis of locally relevant data and existing capacities and resources, we will test whether the program steps can be successfully sustained. However, sustainable and long-term partnerships between schools, communities, and municipalities can be challenging owing to organizational changes or staff turnover. To address this issue, we will seek to engage and motivate commitment at the management level. In addition, the CoP will serve as an informal capacity building and will expectantly keep the organization as the drivers for systems change. If the steps in the program prove to be sustainable and effective, the CoP can be used to disseminate knowledge about methods and approaches to other schools and municipalities interested in implementing the Data Health program.

#### Program Evaluation

This study involves a complex program evaluation. The processes and steps within the Data Health program are systematic but flexible in the sense that the study sites themselves choose the targeted health issue, actions, and process for normalization. The complexity of a systems approach does not allow for a fully randomized controlled trial with a single set of quantitative outcomes [26]. Therefore, we will use a systems-based and quasi-experimental design to test the preliminary effectiveness of the program at an individual and organizational level. We will use mixed methods to account for the complexity and contextual variation. The comprehensive evaluation design of program implementation and outcomes—combining many data sources from different perspectives—will generate new knowledge that can be used in similar or different contexts at the national and international levels.

To our knowledge, few studies have reported on the implementation of systems approaches and sustainment of actions over time [43,77], particularly using GMB as a participatory method. Understanding the use of GMB in a school setting will provide important information for researchers and practitioners regarding enablers and barriers to this approach.

The systems-based evaluation design provides an opportunity to test new system-level evaluation methodologies, such as the Action Scale Model [27], measuring change within complex adaptive systems. There are many uncertainties in studying changes in complex systems, as complex systems are dynamic [26]. Therefore, we recognize that capturing systems change might not be fully comprehensive. To accommodate this, we set systems boundaries to determine what is relevant to capture and will be used to guide the evaluation.

In parallel, we will assess preliminary indications on individual-level outcomes, assessing the effectiveness on students’ health behavior and well-being because of systems change. A limitation of this study is that most of the student data will be cross-sectional because of the vocational school structure. The research team will explore the possibility of tracking individuals’ educational attainment, employment status, and health registers (compiled by Statistics Denmark [78]) 5 or 10 years postprogram implementation.

This study will test a systems change approach in vocational schools and whether such an approach is suitable for adaptation and scale-up. If this study is shown to be sustainable and effective, the established CoP will support implementation at scale.

## Appendix

Multimedia Appendix 1 The Data Health program theory.

## Abbreviations

CLD: causal loop diagram
CoP: community of practice
GMB: Group Model Building
HPS: health promoting school
NCD: noncommunicable disease
RQ: research question
