# Supplementary material for: Developing and Evaluating a Data-Driven and Systems Approach to Health Promotion Among Vocational Students: Protocol for the Data Health Study
Source: JMIR Res Protoc. 2024 Feb 6;13:e52571. doi: 10.2196/52571 (PMC10879971; doi:10.2196/52571)
Supplement: Multimedia Appendix 1 [file resprot_v13i1e52571_app1.pdf]

Multimedia Appendix 1: The Data Health Program theory

This program theory is captured in a series of "if-then" statements – **IF** something is done with or for the program participants, **THEN** something should change (outcome) **BECAUSE** of underlying mechanism(s).

	**IF the activity is provided, THEN what should be the result for participants?**	**WHY do you believe the activity will lead to this result?**	**What evidence do you have that this activity will lead to this result (Middle Rage Theory)?**	**Evaluation indicators** (ways in which this will be measured or assessed)
**Step 1: Collaboration and setup**	IF a formal agreement for collaboration is established between the school and the municipality, THEN the school will be more ready to work with the student's health behavior and wellbeing.	Because managers from both school and municipality support the collaboration and share valuable knowledge.	**Organizational readiness:** Scaccia, J. P et al., *A practical implementation science heuristic for Organizational Readiness: R = MC 2: A Heuristic for Organizational Readiness.* Journal of Community Psychology, 43(4), 484–501.	*Effectiveness:* School organizational readiness<br><br>The role of management support and prioritization
**Step 2: Data-driven approach**	IF a local health profile among the students is carried out and data is presented for students, staff, and school management, THEN the school will experience increased organizational readiness to work with student's health and wellbeing.	Because students, staff and managers gain a shared understanding of the local needs and knowledge about health promotion in general.	**The Icelandic model:** Sigfúsdóttir, I. D., Thorlindsson, T., Kristjánsson, A. L., Roe, K. M., & Allegrante, J. P. (2009). *Substance use prevention for adolescents: The Icelandic Model.* Health Promotion International, 24(1), 16–25.<br><br>**Organizational readiness:** Scaccia, J. P et al., *A practical implementation science heuristic for Organizational Readiness: R = MC 2: A Heuristic for Organizational Readiness.* Journal of Community Psychology, 43(4), 484–501.	*Process evaluation:* Delivery and implementation of planned program components and mechanisms of change<br><br>*Effectiveness:* School organizational readiness

**Step 3: Group model building and cocreation of actions**	IF the systems are mapped, leverage points (where actions are needed) are identified, and relevant people (students, staff, community actors) are involved THEN effective actions for systems change will be implemented.	Because the school allocate resources (time, money etc.).<br><br>Because participants increase their knowledge and engagement to health promotion; and because they work together and share responsibilities and decision making across groups (students, school staff, community actors).	**Group model Building:** Siokou, C., Morgan, R., & Shiell, A. (2014). *Group model building: A participatory approach to understanding and acting on systems.* Public Health Research & Practice, 25(1).<br><br>**Action Scale Model:** Nobles, J. D., Radley, D., Mytton, O. T., & The Whole Systems Obesity program team. (2022). The Action Scales Model: A conceptual tool to identify key points for action within complex adaptive systems. Perspectives in Public Health, 142(6), 328–337**.**	*Process evaluation:* Delivery and implementation of planned program components and mechanisms of change<br><br>*Implementation of actions:* Characteristics of actions
**Step 4: Implementation of actions at different levels**	IF actions are well implemented at different levels (events, structures, goals, beliefs) THEN systems change will occur, and the school will experience increased organizational readiness to work with student health and wellbeing.<br><br>…THEN students will change behavior and experience better health and wellbeing.<br><br>**RIVAL theory**<br><br>IF harmful actions for change are implemented (e.g., actions that promote unhealthy behavior	Because a diverse group of participants (e.g., students, school staff and managers), supported by both municipal and community actors, are highly involved in the implementation process.<br><br>Because students will experience increased health literacy (e.g., knowledge, self-confidence, self-empowerment…) and social mobilization will be initiated (e.g., community competence, social capital, social connectedness…).<br><br>Because the school enforces a healthy environment.	**Action Scale Model:** Nobles, J. D., Radley, D., Mytton, O. T., & The Whole Systems Obesity program team. (2022). The Action Scales Model: A conceptual tool to identify key points for action within complex adaptive systems. Perspectives in Public Health, 142(6), 328–337**.**<br><br>**The Involvement Matrix:** Smits, D.-W., van Meeteren, K., Klem, M., Alsem, M., & Ketelaar, M. (2020). Designing a tool to support patient and public involvement in research projects: The Involvement Matrix. Research Involvement and Engagement, 6(1), 30.<br><br>**Organizational readiness:** Scaccia, J. P et al., *A practical implementation science heuristic for Organizational Readiness: R = MC 2: A Heuristic for Organizational Readiness.* Journal of Community	*Implementation of actions:* Characteristics of actions and related (unknown) mechanisms of change<br><br>*Effectiveness:* School organizational readiness Students' health and wellbeing

	or are too time-consuming for the participants to implement), THEN the school's organizational readiness for working with student's health and wellbeing and student's health behavior and wellbeing will decrease.	Because of some (today unknown) mechanisms that might underlie harm.	Psychology, 43(4), 484–501.<br><br>**Health promotion outcomes:**<br>Bauman AE, Nutbeam D. Evaluation in a nutshell: a practical guide to the evaluation of health promotion programs. 3rd edition. Sydney, NSW: McGraw Hill Education (Australia) Pty Ltd; 2022.	
**Step 5: Normalization and diffusion**	IF the implemented actions, initiated collaborations and organizational practices and motivation are sustained<br>THEN new effective actions for systems change (in the same problem area or in a new health area) are implemented and sustained.<br><br>THEN the school will experience increased organizational readiness and work with health promotion as an integrated part of the core business.	Because the school allocate resources (time, money etc.).<br><br>Because collaborations are established, and the partners experience synergy in the partnership<br><br>Because the representatives from the school and the municipality participate in the community of practice network and share and obtain learnings of implementation and collaborating on effective strategies for health promotion at vocational schools.	**Partnership synergy theory:**<br>Lasker, R. D., Weiss, E. S., & Miller, R. (2001). Partnership Synergy: A Practical Framework for Studying and Strengthening the Collaborative Advantage. The Milbank Quarterly, 79(2), 179–205.<br><br>**Organizational readiness:**<br>Scaccia, J. P et al., *A practical implementation science heuristic for Organizational Readiness: R = MC 2: A Heuristic for Organizational Readiness.* Journal of Community Psychology, 43(4), 484–501.<br><br>**Normalization process theory:**<br>Murray E, Treweek S, Pope C, MacFarlane A, Ballini L, Dowrick C, et al. Normalization process theory: a framework for developing, evaluating, and implementing complex interventions. BMC Med. 2010;8:63.	*Program normalization*: Potentials, barriers and needs for collaborations and program sustainability and mechanisms of change<br><br>*Effectiveness:* School organizational readiness
